# Endogenous HCN Channels Modulate the Firing Activity of Globus Pallidus Neurons in Parkinsonian Animals

**DOI:** 10.3389/fnagi.2019.00190

**Published:** 2019-07-25

**Authors:** Xiao-Meng Hao, Rong Xu, An-Qi Chen, Feng-Jiao Sun, Ying Wang, Hong-Xia Liu, Hua Chen, Yan Xue, Lei Chen

**Affiliations:** ^1^Department of Physiology, Qingdao University, Qingdao, China; ^2^Department of Pathology, Qingdao Municipal Hospital, Qingdao, China

**Keywords:** globus pallidus, hyperpolarization-activated cyclic nucleotide-gated channel, Parkinson’s disease, single unit recording, basal ganglia

## Abstract

The globus pallidus occupies a critical position in the indirect pathway of the basal ganglia motor control system. Hyperpolarization-activated cyclic-nucleotide gated (HCN) channels play an important role in the modulation of neuronal excitability. *In vivo* extracellular single unit recording, behavioral test and immunohistochemistry were performed to explore the possible modulation of endogenous HCN channels in the globus pallidus under parkinsonian states. In MPTP parkinsonian mice, micro-pressure application of the selective HCN channel antagonist, ZD7288, decreased the firing rate in 10 out of the 28 pallidal neurons, while increased the firing rate in another 15 out of the 28 neurons. In 6-OHDA parkinsonian rats, ZD7288 also bidirectionally regulated the spontaneous firing activity of the globus pallidus neurons. The proportion of pallidal neurons with ZD7288-induced slowing of firing rate tended to reduce in both parkinsonian animals. Morphological studies revealed a weaker staining of HCN channels in the globus pallidus under parkinsonian state. Finally, behavioral study demonstrated that intrapallidal microinjection of ZD7288 alleviated locomotor deficits in MPTP parkinsonian mice. These results suggest that endogenous HCN channels modulate the activities of pallidal neurons under parkinsonian states.

## Introduction

Hyperpolarization-activated cyclic nucleotide-gated (HCN) channel family comprises four subunits, HCN1, HCN2, HCN3, and HCN4, which are activated by both membrane hyperpolarization and intracellular cAMP (Biel and Michalakis, 2009; Biel et al., 2009). HCN channels contribute to the spontaneous rhythmic activity of both heart and nervous system. Much evidence indicates that HCN channels play important physiological functions in brain and are responsible for several neurologic diseases (Benarroch, 2013; DiFrancesco and DiFrancesco, 2015), such as epilepsy (Shah et al., 2013), pain (Emery et al., 2011; Resta et al., 2016) as well as Parkinson’s disease (Chan et al., 2004, 2011; Good et al., 2011).

Parkinson’s disease is a neurodegenerative disorder mainly affecting motor systems. Dopaminergic neuron degeneration in the substantia nigra pars compacta gives rise to motor symptoms including akinesia, resting tremor and muscle rigidity. HCN channels are involved in the pathophysiology of Parkinson’s disease. Recent studies reveal the expression and function of HCN channels in nigral dopaminergic neurons (Santoro et al., 2000; Xue et al., 2012; Dufour et al., 2014). In a genetic mitochondrial model of Parkinson’s disease, the HCN channel functions reduce significantly in midbrain dopaminergic neurons (Good et al., 2011). Furthermore, application of 1-Methyl-4-phenylpyridinium (MPP^+^) inhibits the activity of HCN channels in substantia nigra dopaminergic neurons (Masi et al., 2013).

The globus pallidus in rodent is homologous to the external globus pallidus in primates. Being a critical nucleus in the indirect pathway of the basal ganglia circuit, the globus pallidus plays important roles in motor control under both normal and pathological states. In Parkinson’s disease, the depletion of dopamine leads to hypoactivity and synchronized oscillation discharge of globus pallidus neurons, which are closely related to the symptoms of akinesia and resting tremor (Nini et al., 1995; Raz et al., 2000). Morphological studies demonstrate that the globus pallidus expresses a high level of HCN1-4 channels, with HCN2 being the major isoform (Notomi and Shigemoto, 2004). Previous *in vitro* electrophysiological researches revealed that pallidal HCN channels control the spontaneous discharge as well as the response to striatal GABAergic input (Chan et al., 2004). Activation of presynaptic HCN channels decreases GABAergic release in the globus pallidus (Boyes et al., 2007). By using a novel neural network model of globus pallidus neurons, Merrison-Hort and Borisyuk (2013) demonstrate a down-regulation of HCN channels in response to burst firing of pallidal neurons, suggesting the possible involvement of HCN in Parkinson’s disease. Furthermore, acute and chronic dopamine depletion produces a diminished HCN channel activity in the globus pallidus (Chan et al., 2011). Previous studies indicated that the reduced function of HCN channel is likely related to the pathophysiology of Parkinson’s disease (Chan et al., 2011; Merrison-Hort and Borisyuk, 2013). Our previous *in vivo* single unit recordings revealed that HCN channels bidirectionally modulate the spontaneous firing activity of pallidal neurons in normal rats (Chen et al., 2015). In the present study, *in vivo* single unit recording, immunohistochemistry and open field behavioral test were used to further investigate the possible modulation of endogenous HCN channels in the globus pallidus of both 6-OHDA parkinsonian rats and MPTP lesioned mice.

## Materials and Methods

### Animals

In this study, adult male C57BL/6 mice (25–30 g) and adult male Wistar rats (230–310 g) were employed. The mice and rats were kept in polypropylene cages (4 to 5 per cage) under controlled temperature (23 ± 1°C and 50–70% relative humidity). All animals were maintained on a 12:12 h light/dark with standard pellet food and water *ad libitum*. The experiments were approved and all operations referred to institutional guidelines of the Animal Care and Use Committee at Qingdao University. All efforts were taken to minimize animals’ pain or sufferings, and to reduce the amount of animals used. Animals were randomly distributed into various experimental groups.

### Preparation of Parkinsonian Animal Models

To induce parkinsonian mice, MPTP at a dose of 30 mg/kg/day was administrated intraperitoneally for 7 consecutive days. The control group received intraperitoneal treatment with 0.9% normal saline.

To induce 6-OHDA parkinsonian rats, chloral hydrate (400 mg/kg, i.p.) was intraperitoneally injected for anesthesia, and the rats were gently put in a stereotaxic frame (Narishige SN-3, Tokyo, Japan). And then, the skull was exposed and a burr hole was drilled in the skull with 4.3 mm posterior and 1.7 mm lateral to the bregma. A needle of a Hamition microsyringe was introduced into the medial forebrain bundle to a depth of 8.4 mm (Paxinos and Watson, 1998). A total dose of 14.5 μg 6-OHDA hydrochloride (H4381; Sigma, St. Louis, MO, United States) in 4 μl sterile saline containing 0.01% ascorbic acid was then microinjected into the right medial forebrain bundle at a rate of 1.0 μl/min. The needle was left in place for a further 10 min to prevent backflow of the drug. Rats were pretreated 30 min before the 6-OHDA infusion with 25 mg/kg desipramine to protect noradrenergic projections.

Three weeks after the surgery, the rats were subcutaneously received 0.2 mg/kg apomorphine hydrochloride (A4393, Sigma) dissolved in 0.1% ascorbate saline solution. The rats presenting more than 210 contralateral rotations in half an hour were considered successful parkinsonian rats.

### *In vivo* Electrophysiological Recordings

Rats and mice were anesthetized with urethane (1 g/kg, i.p., supplemented as needed) and positioned gently in a stereotaxic frame (Narishige SN-3, Tokyo, Japan, with different head fixing adapters). Rectal temperature was maintained at 36–38°C with a heating pad. According to the stereotaxic atlas, a craniotomy was performed at coordinates of 0.8–1.2 mm posterior and 2.5–3.5 mm lateral from the bregma in rats (Paxinos and Watson, 1998), and 0.3–0.6 mm posterior and 1.7–2.2 mm lateral to the bregma in mice (Franklin and Paxinos, 1997). Three-barrel microelectrodes with the tips of 3–10 mm and resistance of 10–20 MΩ were pulled using a pipette puller (Stoelting, Wood Dale, IL, United States). Then the three-barrel microelectrodes were stereotaxically positioned into the globus pallidus. The recording barrel was filled with 0.5 M sodium acetate containing 2% pontamine sky blue dye. The other two drug ejection barrels contained either ZD7288 or vehicle (normal saline). According to the neuron location and electrophysiological features of a biphasic positive/negative waveform, the globus pallidus neurons were identified for recording. Drug solutions were ejected through four-channel pressure injector (PM2000B, Micro Data Instrument, Inc., United States) with short pulse gas pressure (1500 ms, 5.0–15.0 psi).

The electrical signals were collected with a micro-electrode amplifier (MEZ-8201, Nihon Kohden, Tokyo, Japan), and then displayed on a memory oscilloscope (VC-11, Nihon Kohden). The amplified electrical signals were passed through low and high pass filters between 0.3 and 3 kHz into a bioelectricity signal analyzer and computer. The data was digitized by Micro 1401 and analyzed using spike 2 software (Cambridge Electronic Design, United Kingdom). After stabilization of baseline firing at least 10 min, drug was ejected into the globus pallidus. The basal firing parameters, including firing rate and coefficient of variation (CV) of the interspike intervals (ISI), were determined by the average of 120 s baseline data before drug administration. The drug effect was evaluated by the maximal change of firing values within 120 s after drug application. A significant change in discharge was determined based on the increase or decrease in firing rate exceeded 2 standard deviations (SDs). The CV is defined as the SD of ISI divided by the mean ISI.

### Immunohistochemistry

For immunohistochemical studies, rats and mice (without performing electrophysiological experiments) were deeply anesthetized with intraperitoneal injection of urethane (1.8 g/kg) and then intracardially perfused with 4% paraformaldehyde in phosphate-buffered saline (PBS). The animal brains were rapidly removed and immersed in paraformaldehyde overnight. Then the brains were cryoprotected gradually with 20 and 30% sucrose in 0.1M PBS. After that, brains were frozen and sectioned at 18 μm. Sections containing the globus pallidus were then pre-incubated in diaminobenzidine (DAB) solution, washed in 0.01M PBS 3 times for 5 min and then incubated with 0.3% hydrogen peroxide in order to block the activity of endogenous peroxidase. Then the sections were washed with PBS as previous step and incubated with normal goat serum for 30 min at room temperature. Next, sections were incubated overnight at 4°C in primary antibody solutions as followed: anti-HCN1 antibody (1:300, Millipore) or anti-HCN2 antibody (1:400, Millipore) or anti-HCN3 antibody (1:500, Abcam) and anti-HCN4 antibody (1:300, Millipore). After that, sections were washed in 0.01M PBS 3 times for 5 min to remove unbound antibody and the secondary antibody (HRP conjugated goat anti-rabbit IgG, 1:500; Beijing Zhongshan Biotechnology Co., Beijing, China) was incubated for half an hour at room temperature. The sections were then washed as before and ABC reagent was used for incubation at room temperature for 3 min. Finally, the sections were set at cold water for 5 min. For the quantification analysis of immunostaining, every sixth section was taken throughout the nucleus, with a total of nine sections for each animal. The quantification of positive cells was calculated on five randomly observed visual fields (40 × 10) in each section in a blinded fashion. The positive cells were counted from top left to bottom right using ImageJ software (NIH, United States). The mean value of each section was obtained from all the observed visual fields of one animal.

To determine the lesions of nigral dopaminergic neurons, sections containing the substantia nigra pars compacta were incubated overnight at 4°C with monoclonal anti-tyrosine hydroxylase antibody (1:5,000; Sigma). In 6-OHDA parkinsonian rats, the percentage of tyrosine hydroxylase-positive neuronal loss was 95.11 ± 1.07%. In MPTP parkinsonian mice, the percentage of tyrosine hydroxylase-positive neuronal loss was 68.31 ± 2.79% (Supplementary Figure 1).

### Open Field Test in Mice

The open field activity monitoring system comprehensively assesses locomotor activity levels of mice. The test is also widely used to assess the efficacy of therapeutic drugs that may improve locomotion and/or motor function (Malerba et al., 2011; Yu et al., 2013). The mice were anesthetized with 8% chloral hydrate (400 mg/kg, i.p.). According to the stereotaxic atlas, two stainless steel guide cannulae (outside diameter 0.4 mm, inside diameter 0.3 mm) were bilaterally implanted into the globus pallidus (0.3 mm posterior to bregma, 1.8 mm lateral lateral to the midline, and 4.0 mm ventral from the skull surface). And then, the cannulae were fixed to the skull with stainless steel screw and dental acrylic. After 5 days of recovery, open field test was conducted. Twenty minutes before the test, the mice received bilateral infusion of saline or 0.05 mM ZD7288 (0.3 μl for each side) into the globus pallidus. Each mouse was placed within the center of the square arena (27.3 × 27.3 × 20.3 cm). All open field tests were performed between 9:00 am and 12:00 am. Total distance traveled (cm) over 60 min and velocity (cm/s) were analyzed.

### Histological Controls

After electrophysiological experiments, the pontamine sky blue was employed to verify the position of the recording site of three-barreled microelectrodes using iontophoresis technique (10 μA, 20 min). After behavioral tests, the mice were deeply anesthetized with chloral hydrate (600 mg/kg, i.p.). All rats and mice used in the experiments were anesthetized and intracardially perfused with 4% paraformaldehyde solution. The brains were removed and then sectioned at 50 μm with a freezing microtome. All the recording and microinjection sites were identified under light microscope (Supplementary Figure 2). Only the animals with correct positions were included for further data analysis.

### Drugs and Statistics

ZD7288 was obtained from Tocris (Bristol, United Kingdom). 6-OHDA hydrochloride, MPTP and apomorphine hydrochloride were purchased from Sigma (St. Louis, MO, United States). Rabbit anti-HCN1 (AB5884), anti-HCN2 (AB5378) and anti-HCN4 (AB5808) polyclonal antibodies were obtained from Millipore (Massachusetts, United States). Rabbit anti-HCN3 antibodies (ab65705) were obtained from Abcam (Cambridge, United Kingdom). Histostain TM-Plus Kits used in DAB immunohistochemical staining was obtained from ZSGB-BIO (Beijing, China).

The data were expressed as mean ± SEM. Paired *t*-test was used to compare the difference of firing rate or CV before and after drug application. Student’s *t*-test and one-way ANOVA were used to analyze statistical comparisons between or among groups. Before *t*-tests or ANOVA, Levene’s test was performed to determine if the data is normally distributed. Chi-Square test was used to compare the fractions of different firing pattern types between normal and parkinsonian animals. The level of significance was presented by using a *P*-value of 0.05.

## Results

### Firing Characteristics of Globus Pallidus Neurons in Mice

**FIGURE 1:**
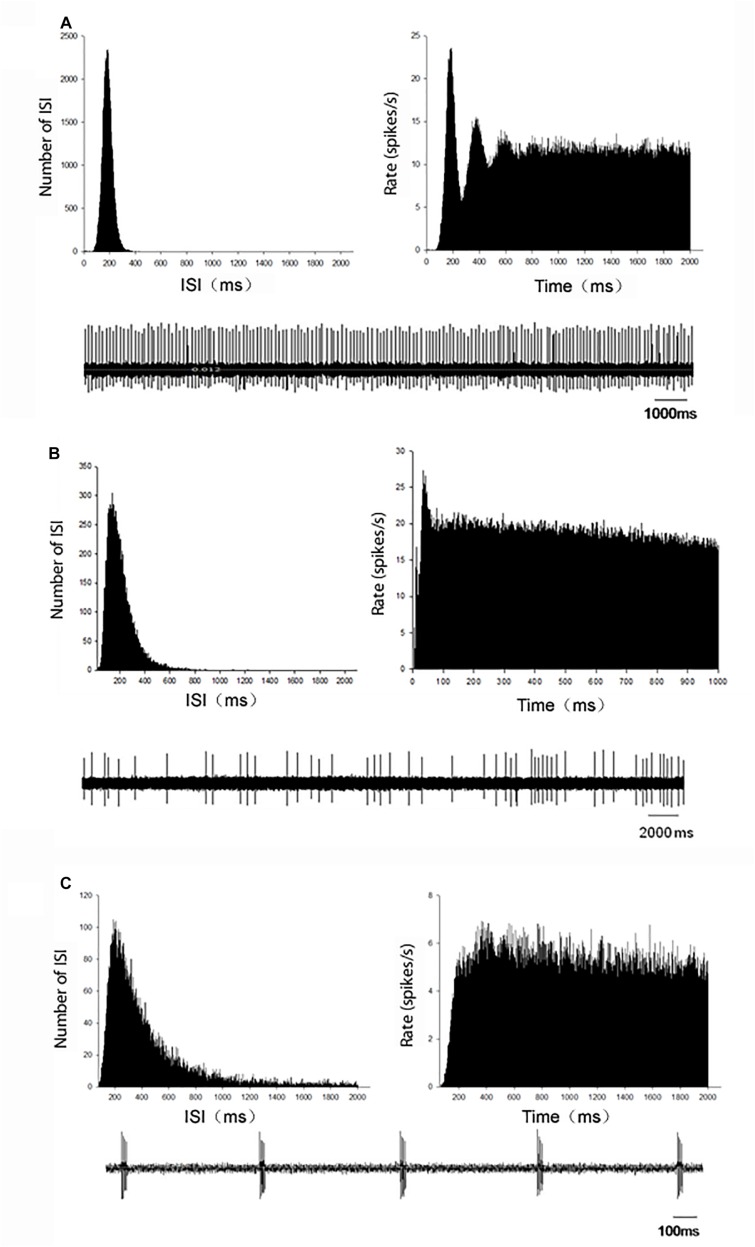
Representative firing patterns of the globus pallidus neurons in C57BL/6 mice. According to the ISI histogram and autocorrelogram, three different firing types, regular **(A)**, irregular **(B)**, and burst **(C)**, were acquired from the globus pallidus neurons. Left upper trace: ISI histogram; Right upper trace: autocorrelogram; Low trace: original firing.

In MPTP parkinsonian mice, total 40 globus pallidus neurons were recorded. The average firing rate was 7.77 ± 0.77 Hz. In present recording of pallidal neurons in normal mice with normal saline injection intraperitoneally, the average spontaneous firing rate was 9.27 ± 1.40 Hz (*n* = 34), which was not significantly different from that in MPTP parkinsonian mice (*P* > 0.05). Based on the ISI histogram and autocorrelogram, three firing types of pallidal neurons were classified, regular firing neurons, irregular firing neurons and burst firing neurons (Figure 1). Table 1 showed the comparison of the three firing pattern neurons between normal and MPTP parkinsonian mice. The fraction of burst firing neurons in MPTP parkinsonian mice was more than that in normal mice (*P* < 0.05).

**TABLE 1:** Comparison of the firing patterns between normal and MPTP parkinsonian mice.

**Group**	**Regular firing neurons**		**Irregular firing neurons**		**Burst firing neurons**		**Total**
	**(*n*)**	**(%)**	**(*n*)**	**(%)**	**(*n*)**	**(%)**	**(*n*)**
Normal mice	11	32.4%	16	47.1%	7	20.5%	34
MPTP mice	8	20.0%	18	45.0%	14	35.0%^*^	40

^*^P < 0.05 compared with that of normal mice.

### Electrophysiological Effects of ZD7288 on the Spontaneous Firing of Globus Pallidus Neurons in MPTP Parkinsonian Mice

As a control, we firstly observed the effects of ZD7288 in normal mice intraperitoneally injected with normal saline. During 34 neurons recorded, micro-pressure ejection of 0.05 mM ZD7288 decreased the frequency of spontaneous firing from 10.4 ± 2.1 Hz to 5.0 ± 1.2 Hz in 17 out of the 34 (50.0%) pallidal neurons (*n* = 17, *P* < 0.001). The average decrease was 55.2 ± 4.5%, which was significantly different (*P* < 0.001) compared to that of vehicle ejection (basal: 7.6 ± 2.1 Hz; vehicle: 7.2 ± 1.4 Hz; *n* = 8, *P* > 0.05). In another 15 (44.1%) pallidal neurons, intrapallidal injection of ZD7288 increased the firing rate from 6.8 ± 1.9 Hz to 12.1 ± 3.0 Hz with the average increase of 108.1 ± 17.1% (*P* < 0.001). ZD7288 had no affects on the remaining 2 neurons.

**FIGURE 2:**
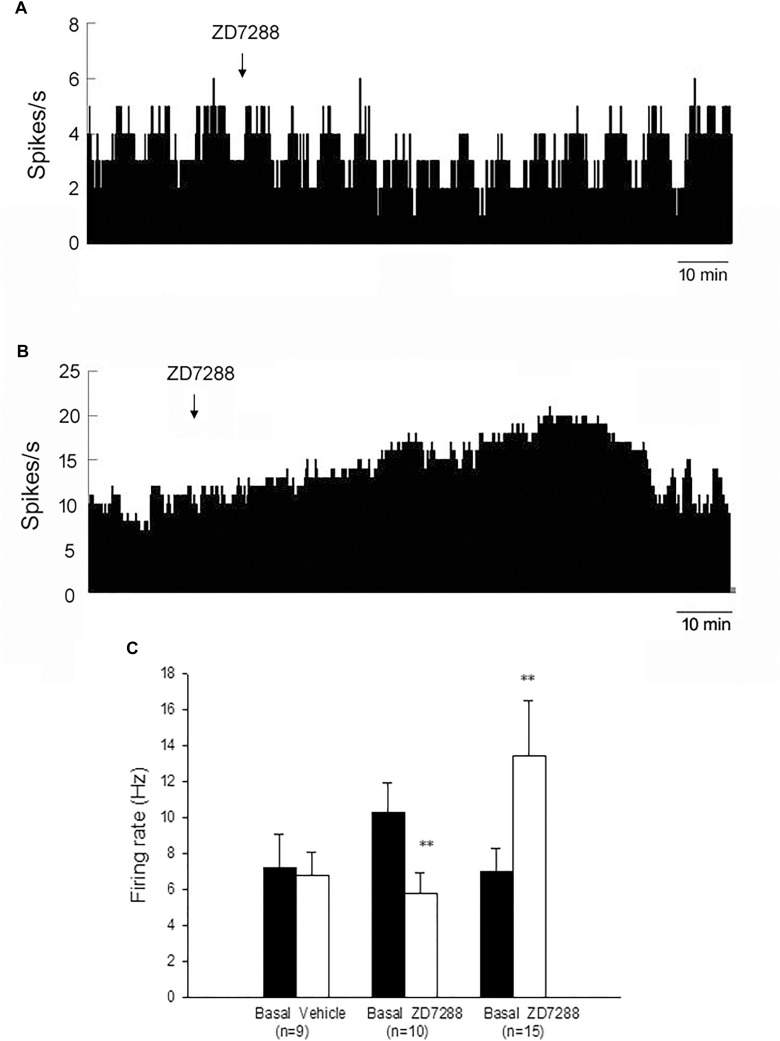
HCN channels bidirectionally modulated the spontaneous firing rate of the globus pallidus neurons in MPTP parkinsonian mice. **(A)** Typical frequency histograms presenting that ZD7288 reduced the firing rate of a globus pallidus neuron. **(B)** In this neuron, ZD7288 increased the firing rate. **(C)** Pooled data summarizing the effects of ZD7288 on the spontaneous firing rate of the globus pallidus neurons. ^∗∗^*P* < 0.01 compared to basal firing.

In MPTP parkinsonian mice, we observed the electrophysiological effects of ZD7288 completely in only 28 out of the total 40 pallidal neurons. Micro-pressure ejection of 0.05 mM ZD7288 decreased the firing rate from 10.3 ± 1.6 Hz to 5.8 ± 1.1 Hz in 10 out of the 28 (35.7%) pallidal neurons (*n* = 10; *P* < 0.01; Figures 2A,C). The average decrease was 40.4 ± 6.1%. In another 15 (53.6%) pallidal neurons, ZD7288 increased the spontaneous firing rate from 7.0 ± 1.3 Hz to 13.4 ± 3.1 Hz (*n* = 15; *P* < 0.01; Figures 2B,C). The average increase was 150.9 ± 53.7%. In the remaining three neurons, ZD7288 did not change the firing rate significantly. In 9 out of the 28 pallidal neurons, normal saline was administrated prior to ZD7288 as control. Normal saline did not change the firing rate significantly (basal: 7.2 ± 1.9 Hz, normal saline: 6.8 ± 1.3 Hz; *n* = 9; P > 0.05; Figure 2C). We analyzed the possible correlation between ZD7288-induced effects on neuronal activity and the different firing patterns. The results showed that ZD7288 exerted similar effects on different firing patterns of pallidal neurons (*P* > 0.05). The results were shown in Table 2.

**TABLE 2:** Comparison of the role of ZD7288 in pallidal neurons with different firing patterns in MPTP parkinsonian mice.

**Group**	**Regular firing eurons**		**Irregular firing neurons**		**Burst firing neurons**		**Total**
	**(*n*)**	**(%)**	**(*n*)**	**(%)**	**(*n*)**	**(%)**	**(*n*)**
Excitatory response	4	26.7	7	46.6	4	26.7	15
Inhibitory response	1	10.0	5	50.0	4	40.0	10

*Fisher’s exact test *P* = 0.758.*

Next, we analyzed whether ZD7288 changes the CV of pallidal neurons. In the neurons with ZD7288-induced decrease of firing rate, ZD7288 did not change the CV significantly (basal: 0.58 ± 0.16, ZD7288: 0.85 ± 0.29, *n* = 10; *P* > 0.05). In the neurons with ZD7288-induced increase of firing rate, ZD7288 did not change the CV significantly (basal: 0.66 ± 0.12, ZD7288: 0.78 ± 0.19; *n* = 15; *P* > 0.05) too.

The effects of ZD7288 in the globus pallidus were compared between normal and MPTP treated mice. There was no statistical difference for ZD7288-induced decrease in firing rate between normal (55.2 ± 4.5%) and MPTP parkinsonian mice (40.4 ± 6.1%, *P* > 0.05). Although ZD7288 tended to induce a stronger excitatory effect in MPTP parkinsonian mice (150.9 ± 53.7%) compared to that of normal group (108.1 ± 17.1%), there was no statistical difference either (*P* > 0.05). Furthermore, the proportion of pallidal neurons with ZD7288-induced slowing of firing rate (35.7%) in parkinsonian mice tended to be less than that of normal mice (50.0%).

### Electrophysiological Effects of ZD7288 on the Spontaneous Firing of Globus Pallidus Neurons in 6-OHDA Parkinsonian Rats

To clarify the modulation of HCN channels on globus pallidus neurons in 6-OHDA-lesioned parkinsonian rats, we monitored the spontaneous firing activity of 55 pallidal neurons in 6-OHDA parkinsonian rats. The average spontaneous firing rate was 8.4 ± 1.3 Hz (*n* = 27) on the lesioned side, and 12.4 ± 1.7 Hz (*n* = 28) on the unlesioned side. The firing rate on the lesioned side tended to be lower than that on the unlesioned side but there was no statistical difference (*P* = 0.07). Usually, we could record 3–5 pallidal neurons on the unlesioned side of 6-OHDA parkinsonian rats or each side of the globus pallidus neurons in normal rats. However, in parkinsonian rats, at most one pallidal neuron was recorded on the lesioned side. Sometimes, we could not even record one pallidal neuron with spontaneous firing.

**FIGURE 3:**
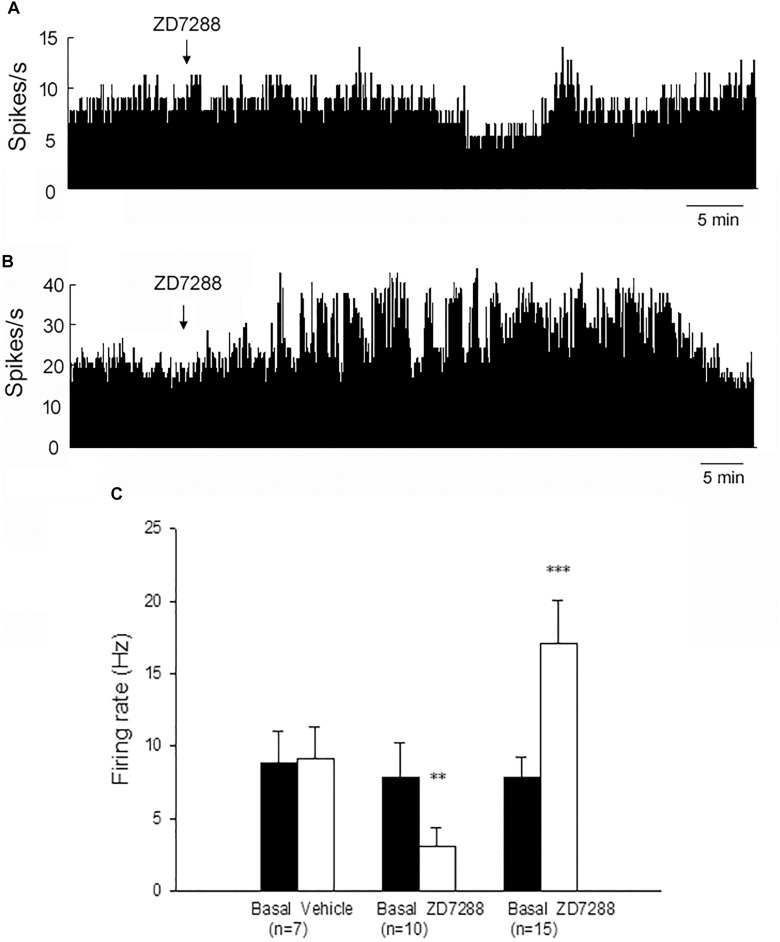
Modulation of the spontaneous firing rate by HCN channels on the globus pallidus neurons in 6-OHDA parkinsonian rats. Typical frequency histograms showing that ZD7288 decreased **(A)** or increased **(B)** the firing rate of globus pallidus neurons in 6-OHDA parkinsonian rats. **(C)** Pooled data summarizing the effects of vehicle and ZD7288 on the spontaneous firing rate of the globus pallidus neurons. ^∗∗^*P* < 0.01, ^∗∗∗^*P* < 0.001 compared to basal firing rate.

On the lesioned side, local administration of ZD7288 decreased the firing rate from 7.8 ± 2.4 Hz to 3.1 ± 1.3 Hz in 10 out of the 27 (37.0%) pallidal neurons (*n* = 10; *P* < 0.01; Figures 3A,C). The average decrease was 70.2 ± 7.5%. In another 15 out of the 27 (55.6%) pallidal neurons, ZD7288 increased the spontaneous firing rate from 7.8 ± 1.4 Hz to 17.1 ± 3.0 Hz (*n* = 15; *P* < 0.001; Figures 3B,C). The average increase was 149.8 ± 29.8%. ZD7288 did not significantly alter the firing rate in remaining 2 pallidal neurons. In 7 pallidal neurons, normal saline was administrated prior to ZD7288 as control. Normal saline did not change the firing rate significantly (basal: 8.8 ± 2.2 Hz, normal saline: 9.1 ± 2.2 Hz; *n* = 9; *P* > 0.05; Figure 3C). Furthermore, on the unlesioned side of 6-OHDA parkinsonian rats, ZD7288 significantly decreased the firing rate by 46.1 ± 4.6% in 12 out of the 28 (42.9%) neurons (basal: 12.8 ± 2.4 Hz, ZD7288: 7.1 ± 1.4 Hz, *n* = 12; *P* < 0.001). In 10 out of the 28 (35.7%) neurons, ZD7288 increased the firing rate by 130.7 ± 34.2% (basal: 7.9 ± 2.4 Hz, ZD7288: 14.3 ± 3.0 Hz; *n* = 10; *P* < 0.001).

We further analyzed ZD7288-induced change of firing rate between 6-OHDA-lesioned side and unlesioned side of parkinsonian rats. Statistical tests showed that ZD7288-induced decrease in firing rate on the lesioned side of parkinsonian rats was significantly stronger than that on the unlesioned side (*P* = 0.010). There was no statistical difference for ZD7288-induced increase in firing rate between 6-OHDA-lesioned side and unlesioned side (*P* > 0.05).

### Expression of HCN1, HCN2, HCN3, and HCN4 Subtypes on the Globus Pallidus Neurons of Both Normal and Parkinsonian Animals

**FIGURE 4:**
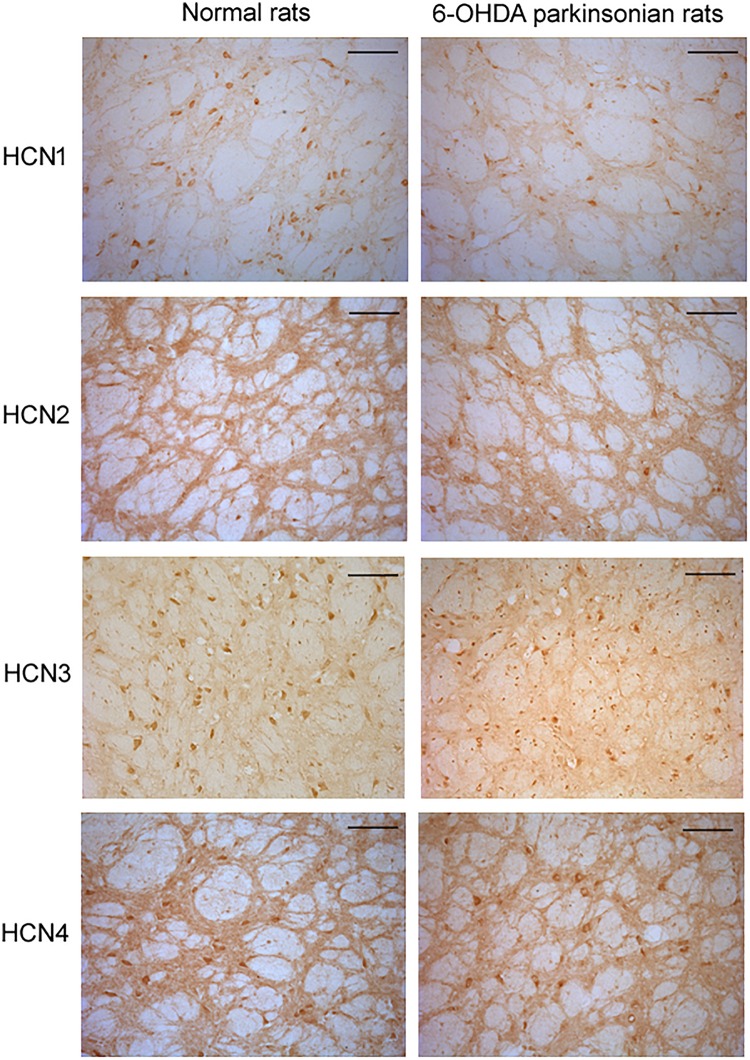
Expression of HCN channels in the globus pallidus of both normal and 6-OHDA parkinsonian rats. Immunolabeling for HCN1 was observed mainly in the cell body and weakly in the neuropil. However, strong immunolabeling for HCN2 was observed in the neuropil, with less perikaryal labeling. Immunolabeling for HCN3 was observed in both cell body and neuropil elements in the globus pallidus. Immunolabeling for HCN4 was also present in the globus pallidus, with similar strength of labeling in cell body and neuropil elements. Scale bars: 100 μm.

In the globus pallidus neurons of both normal and 6-OHDA-lesioned rats, positive immunolabeling for HCN1, HCN2, HCN3 and HCN4 was observed in the medium-size multipolar neurons (Figure 4). Immunolabeling for HCN1 was mainly observed in the cell body and weakly in the neuropil. However, the perikaryal labeling for HCN2 was less intense than HCN1, but stronger HCN2 labeling was observed in the neuropil. Immunolabeling for HCN3 was observed mainly in the cell bodies of the globus pallidus neurons. Moderate immunolabeling for HCN4 was present in the globus pallidus with similar strength of labeling on the cell bodies and neuropil elements. In 6-OHDA parkinsonian rats, quantification analysis showed that the number of positive cells of HCN1 channel subtype (7.32 ± 1.06, *n* = 6) was less than that in normal rats (11.66 ± 1.14, *n* = 6, *P* < 0.05). The number of positive cells of HCN3 channel subtype (9.50 ± 1.04, *n* = 7) was also less than that in normal rats (13.67 ± 1.36, *n* = 7, *P* < 0.05). Similarly, the HCN2 immunopositive neuropil in parkinsonian rats was weaker than that of normal rats.

**FIGURE 5:**
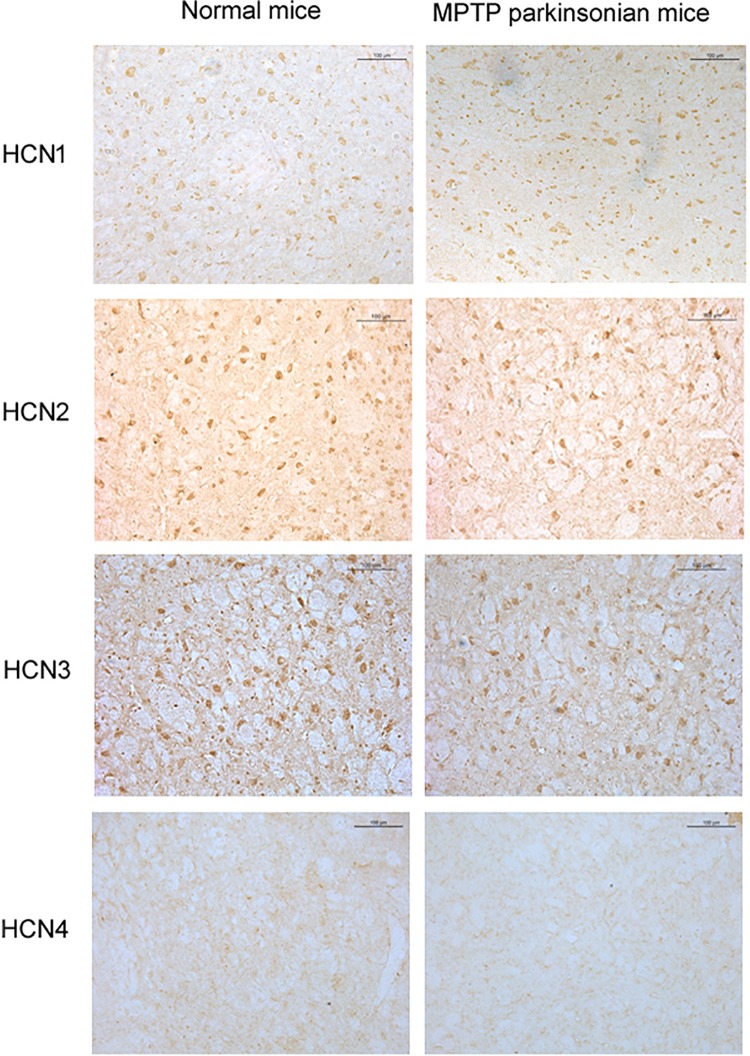
Expression of HCN channels in the globus pallidus of both normal and MPTP parkinsonian mice. Strong positive immunolabeling for HCN2 and HCN3 was observed in the cell body and neuropil. Moderate HCN1 was observed mainly in the cell body. Very weak HCN4 immunostaining was observed in the neuropil. Scale bars: 100 μm.

Positive expression of HCN1, HCN2, HCN3, and HCN4 subtypes was detected in the globus pallidus of both normal and MPTP parkinsonian mice (Figure 5). Moderate HCN1 channel subtype was observed mainly in the cell bodies of globus pallidus neurons. Strong HCN2 and HCN3 channel subtypes were observed on both cell body and neuropil elements. HCN4 channel subtype was rarely observed on neuropil elements. In MPTP parkinsonian mice, quantification analysis showed that the number of positive cells of HCN2 channel subtype was 8.70 ± 1.58 (*n* = 10), which was less than that in normal mice (10.69 ± 2.49, *n* = 9, *P* < 0.05). In addition, the number of positive cells of HCN3 channel subtype (7.25 ± 0.88, *n* = 7) was also less than that in normal mice (10.08 ± 1.13, *n* = 9, *P* < 0.001). As for the number of HCN1 positive neurons, there was no significant difference between parkinsonian mice (8.72 ± 1.76, *n* = 9) and normal mice (10.97 ± 2.61, *n* = 9, *P* > 0.05).

### Intrapallidal Infusion of ZD7288 Alleviate Locomotor Deficits in MPTP Parkinsonian Mice

**FIGURE 6:**
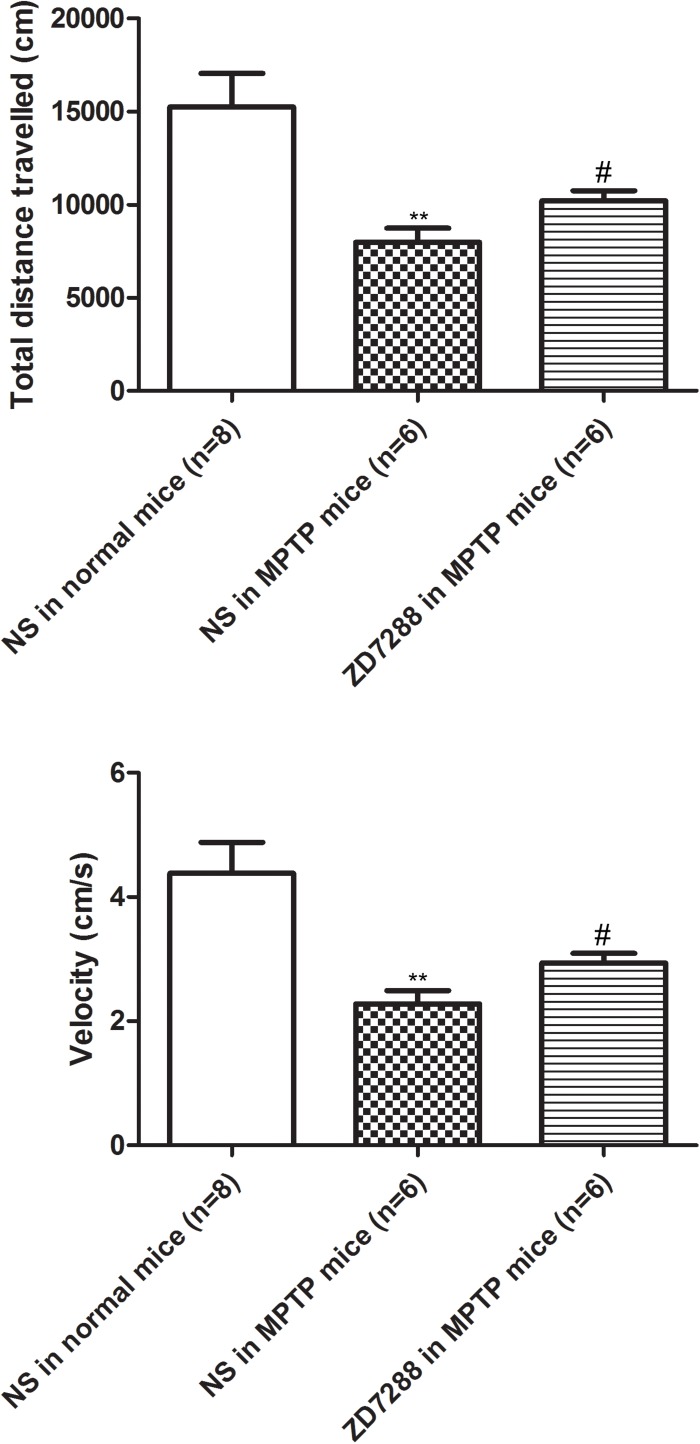
Effects of bilateral microinjection of ZD7288 into the globus pallidus on motor behavior in open field test. Open field analysis showing that locomotor behavior was decreased in MPTP parkinsonian mice. Intrapallidal infusion of ZD7288 alleviated motor deficits in MPTP parkinsonian mice. ^∗∗^*P* < 0.01 compared to that of normal saline in normal mice; ^#^*P* < 0.05 compared to that of normal saline in MPTP treated mice. NS, normal saline.

To provide evidence that endogenous HCN channels in the globus pallidus modulate locomotor activity, the effects of ZD7288 on motor behaviors were studied in awake animals. As shown in Figure 6, in normal saline group of MPTP parkinsonian mice, the total distance moved and velocity were 7979.20 ± 751.96 cm and 2.28 ± 0.21 cm/s, respectively (*n* = 6), which was significantly decreased compared to that of normal saline group in normal mice (total distance: 15236.54 ± 1801.07 cm, velocity: 4.38 ± 0.49 cm/s, *n* = 8, *P* < 0.01 respectively). In intrapallidal application of ZD7288 group in MPTP parkinsonian mice, the total distance moved and velocity were 10185.21 ± 554.86 cm and 2.94 ± 0.16 cm/s (*n* = 6), which was significantly improved compared to that of normal saline group in MPTP treated mice (*P* < 0.05).

## Discussion

By using *in vivo* single unit recordings, we reported here that the basal firing rate of the globus pallidus neurons in adult C57BL/6 mice was around 8 Hz. Three firing patterns of pallidal neurons, regular, irregular and burst were classified in mice. In MPTP parkinsonian mice, the basal firing rate of the recorded globus pallidus neurons was not significantly different from that of normal mice. Furthermore, the fraction of burst firing neurons in MPTP parkinsonian mice was more than that in normal mice. The basal firing rate of pallidal neurons on the lesioned side of 6-OHDA parkinsonian rats tended to be slower than that on the unlesioned side but there was no statistical difference.

Previous studies indicated that the firing rate of pallidal neurons decreases in 6-OHDA lesioned parkinsonian rats (Chang et al., 2006) and parkinsonian monkey (Soares et al., 2004). However, the present *in vivo* recordings showed that the basal firing rate of the globus pallidus neurons in 6-OHDA-induced parkinsonian rats was not significantly different compared to that of normal rats. One of the possibilities of the discrepancy on the firing rate change under parkinsonian state may be caused by the anesthesia. The firing rate of pallidal neurons decreases in freely moving parkinsonian rats or monkey (Soares et al., 2004; Chang et al., 2006), but does not change in anesthetized animals (Breit et al., 2007; Xue et al., 2009) including the present parkinsonian animals. However, studies by Mallet et al. (2008) revealed that the average firing rate of all globus pallidus neurons under parkinsonian state decreases significantly during both slow-wave activity and cortical activation rats. Another possible reason is the stage of Parkinson’s disease. A study by Breit et al. (2007) demonstrated that in advanced stage model of Parkinson’s disease (microinjection of 6-OHDA into the SNc), the mean firing rate of globus pallidus neurons does not change compared to normal controls. However, in early stage model of the disease (microinjection of 6-OHDA into the striatum), the globus pallidus neurons show a significant reduction of firing rate.

Recently two main types of pallidal neurons, PV^+^ and Npas1^+^ neurons, are identified based on their molecular and physiological properties (Hernandez et al., 2015). The PV^+^ neurons and Npas1^+^ neurons account for about 55 and 27% globus pallidus neurons, respectively. It is demonstrated that the Npas1^+^ neurons fire at relatively slower rate than PV^+^ neurons. In chronic 6-OHDA lesioned parkinsonian mice, the spontaneous firing activity of Npas1^+^ neurons decrease significantly, while the firing rates of PV^+^ neurons is preserved. As the proportion and basal firing rate of Npas1^+^ neurons are relatively lower than that of PV^+^ neurons, we therefore hypothesized that the selective decrease of firing rate of Npas1^+^ neurons might not influence the total average firing rate of the globus pallidus neurons under parkinsonian states.

HCN channels function as primary pacemaker in many brain regions (Pape and McCormick, 1989; Maccaferri and McBain, 1996; Bal and McCormick, 1997). Previous *in vitro* electrophysiological recordings revealed that ZD7288 significantly decreases the spontaneous firing of pallidal neurons, suggesting the involvement of HCN channels in autonomous firing of the globus pallidus neurons (Chan et al., 2004). Our previous *in vivo* electrophysiological studies revealed that endogenous HCN channels play important roles in autonomous firing of the globus pallidus neurons in normal animals. Blockade of HCN channels bidirectionally modulated the spontaneous firing activity of globus pallidus neurons (Chen et al., 2015). Similar to that of normal animals, blockade of HCN channels by ZD7288 also produced either decrease or increase of the spontaneous firing of the globus pallidus neurons in both MPTP parkinsonian mice and 6-OHDA parkinsonian rats. It is well known that HCN channels are “pacemaker channels” as they help to generate rhythmic activity within neurons. Application of ZD7288 blocked HCN channels and therefore significantly reduced the spontaneous discharge rate of pallidal neurons. However, in addition to decreasing the spontaneous firing, our *in vivo* extracellular recording revealed that ZD7288 increased the firing rate in partial globus pallidus neurons. Similar ZD7288-induced increase in baseline firing rate was reported in ventral tegmental area and prelimbic cortex (McDaid et al., 2008; Li et al., 2010). It is known that HCN channels are involved in the integration of dendritic excitatory glutamatergic synaptic inputs in a variety of neurons. Activation of HCN channels reduce the amplitude and time course of excitatory postsynaptic potentials (Williams and Stuart, 2000; Angelo et al., 2007; Bullis et al., 2007; Ying et al., 2007; Masi et al., 2013). As our previous studies in normal rats, the interaction of HCN channels with glutamatergic transmission may be one of the possible reasons in ZD7288-induced increase in firing rate (Chen et al., 2015). There are abundant axon collaterals between globus pallidus neurons, which may interrupt the basal discharge of pallidal neurons by GABA release (Chan et al., 2004). Previous studies have shown that blockade of HCN channels by ZD7288 modulates GABAergic neurotransmission in some brain regions including the globus pallidus, subthalamic nucleus, hippocampus and cerebellum (Southan et al., 2000; Aponte et al., 2006; Boyes et al., 2007; Atherton et al., 2010). Therefore, in addition to postsynaptic HCN channels, the present ZD7288-induced modulation of firing rate may be related to the modulation of excitatory and inhibitory synaptic transmission within the globus pallidus neurons.

HCN channels are encoded by four genes (HCN1, 2, 3, 4) and are widely expressed throughout the central nervous system. Present immunohistochemical staining revealed the positive expression of HCN1, HCN2, HCN3, and HCN4 subtypes in the globus pallidus neurons. Previous morphological studies have shown that both HCN1 and HCN2 subtypes are located in somata and dendritic processes, axon and axon terminals of the globus pallidus, predominantly on axon terminals (Boyes et al., 2007). Different from previous studies, it is revealed that the present immunostaining of HCN1 was located mainly in the cell body and weakly in the neuropil. This discrepancy could be due to the different antibodies and rat species used. The present bidirectional modulation of the firing activity by HCN channels may also be produced by its activity on different subtypes of HCN channels in the globus pallidus neurons.

Studies by Chan et al. (2011) revealed that dopamine depletion produces a progressive loss of autonomous pacemaking in globus pallidus neurons and the loss is resulted from the down-regulation of HCN channels. In a genetic mitochondrial model of Parkinson’s disease, a large reduction in HCN channel function was observed in midbrain dopamine neurons (Good et al., 2011). Therefore, HCN channels in the basal ganglia may have a relationship with Parkinson’s disease. It is well known that HCN channels determine the rate and regularity of spontaneous spike activity of the globus pallidus neurons. The present electrophysiological recordings showed that the basal firing rate of globus pallidus neurons in parkinsonian animals tended to be lower compared to that of normal animals. Furthermore, in both parkinsonian rats and mice recorded in present study, it was not easy to find a pallidal neuron with spontaneous firing, which may suggest that some pallidal neurons became silent under parkinsonian state. In line with present electrophysiological recordings, Chan et al. (2011) reported that ∼60% of globus pallidus neurons lose their normally robust autonomous pacemaking in parkinsonian animals. All these suggested that the possible down-regulation of HCN channels under parkinsonian state may be partially involved in the present lower basal firing rate of globus pallidus neurons as well as the fewer firing neurons. In addition to electrophysiological results, present immunostaining showed the decreased expression of HCN subtypes in the globus pallidus of parkinsonian animals. Consistently, by using Western blot analysis, Chan et al. (2011) demonstrated that dopamine depletion with reserpine decreases the expression of all four HCN α-subunits (HCN1–4) in the globus pallidus. Further quantitative real-time PCR reveals a significant reduction in the abundance of all four HCN subunit mRNAs under parkinsonian states.

In present *in vivo* extracellular recordings, blockade of HCN channels by ZD7288 decreased the spontaneous firing rate in 35.7% (10 out of 28) and 37.0% (10 out of 27) pallidal neurons in parkinsonian mice and rats, respectively. In normal animals, ZD7288 decreased the firing rate in 50.0% (17 out of 34) and 42.9% (12 out of 28) pallidal neurons in normal mice and rats, respectively. The tendency of reduced proportion of ZD7288-induced slowing of firing rate may reflect the possible down-regulation of HCN channels in the control of pacemaker activity of pallidal neurons under parkinsonian states. Consistent with the electrophysiological study, present morphological results revealed weaker immunostainings for HCN subtypes under parkinsonian states. However, the present electrophysiological recordings revealed that ZD7288-induced decrease of firing rate on the lesioned side (70.2%) of 6-OHDA parkinsonian rats was stronger than that on the unlesioned side (46.1%). We did not know the exact reasons for the enhancement of ZD7288-induced decrease of firing rate in parkinsonian rats. It is known that HCN channel blocker ZD7288 increases GABA release in the globus pallidus (Boyes et al., 2007). Under parkinsonian state, the depletion of dopamine leads to overactivity of the striatopallidal GABAergic innervation. One possibility may be that ZD7288 induces more GABA release and therefore exerts stronger inhibition in 6-OHDA parkinsonian rats.

The present open field test showed that intrapallidal injection of ZD7288 significantly improved locomotor activity in MPTP parkinsonian mice. The behavioral test suggested that the excitatory effects of ZD7288 may exceed its inhibitory action on pallidal neurons. The possible explanation may be as follows: (1) The globus pallidus mainly receives glutamatergic inputs from the subthalamic nucleus, and GABAergic inputs from the striatum and local axon collaterals (Smith et al., 1998; Sadek et al., 2007). Under parkinsonian state, the globus pallidus receives more excitatory afferents for the abnormal hyperactivity of the subthalamic nucleus. (2) Morphological study demonstrated that HCN2 and to a lesser degree HCN1 was observed in glutamatergic axon terminals of the globus pallidus (Boyes et al., 2007). Considering HCN channels inhibit glutamate synaptic release at select cortical synapses (Huang et al., 2011), we supposed that ZD7288 might more strongly blocked this inhibitory effects on glutamate release under parkinsonian state. (3) Our electrophysiological recordings also revealed that the excitatory effect of ZD7288 tended to be stronger in MPTP parkinsonian mice. As a result, the resulting hyperactivity of the globus pallidus disinhibits the basal ganglia targets leading to the improvement of locomotor activity.

In summary, the present single unit recording revealed that blockade of HCN channels by ZD7288 exerted either inhibition or excitation on the spontaneous firing rate of globus pallidus neurons under parkinsonian states. The reduced proportion of pallidal neurons with ZD7288-induced slowing of firing rate in both parkinsonian mice and rats may suggest the possible down-regulation of HCN channels. The present electrophysiological, morphological, and behavioral studies provide further evidence for the involvement of pallidal HCN channels in Parkinson’s disease.

## Data Availability

All datasets generated for this study are included in the manuscript and/or the Supplementary Files.

## Ethics Statement

This study was carried out in accordance with the recommendations of ’institutional guidelines of the Animal Care and Use Committee at Qingdao University. All efforts were taken to minimize animals’ pain or sufferings and to reduce the amount of animals applied. The protocol was approved by the Animal Care and Use Committee at Qingdao University.

## Author Contributions

X-MH, RX, A-QC, F-JS, and YW performed the electrophysiological experiments in MPTP treated mice and 6-OHDA parkinsonian rats. H-XL and HC conducted the expression of HCN subtypes on the globus pallidus neurons of both normal and parkinsonian animals. YX performed the behavior tests in MPTP parkinsonian mice. YX and LC designed the research, supplied the experiments idea, analyzed the data, and wrote the manuscript.

## Conflict of Interest Statement

The authors declare that the research was conducted in the absence of any commercial or financial relationships that could be construed as a potential conflict of interest.

## Supplementary Material

FIGURE S1 Immunostaining of tyrosine hydroxylase (TH) in the substantia nigra pars compacta of both normal and parkinsonian animals. The number of TH-positive neurons in the substantia nigra pars compacta of both MPTP parkinsonian mice and 6-OHDA parkinsonian rats were significantly less than that of normal animals. Scale bars: 500 μm.

FIGURE S2 Histological verification of the recording sites. Typical photomicrographs of the coronal sections revealing the traces of microelectrodes (arrow pointing) were confined to the globus pallidus in mice **(A)** and rats **(B)**. Str, striatum; GP, globus pallidus. Scale bars: 500 μm **(A)**, 1 mm **(B)**.
