# Supplementary figures and images for: Endogenous HCN Channels Modulate the Firing Activity of Globus Pallidus Neurons in Parkinsonian Animals
Source: Front Aging Neurosci. 2019 Jul 25;11:190. doi: 10.3389/fnagi.2019.00190 (PMC6670024; doi:10.3389/fnagi.2019.00190)

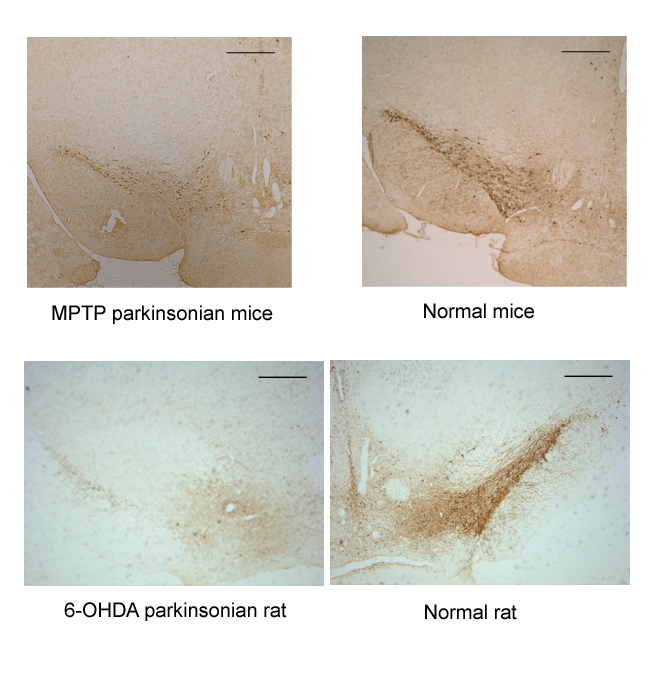

Supplement: FIGURE S1 — Immunostaining of tyrosine hydroxylase (TH) in the substantia nigra pars compacta of both normal and parkinsonian animals. The number of TH-positive neurons in the substantia nigra pars compacta of both MPTP parkinsonian mice and 6-OHDA parkinsonian rats were significantly less than that of normal animals. Scale bars: 500 μm. [file Image_1.TIF]

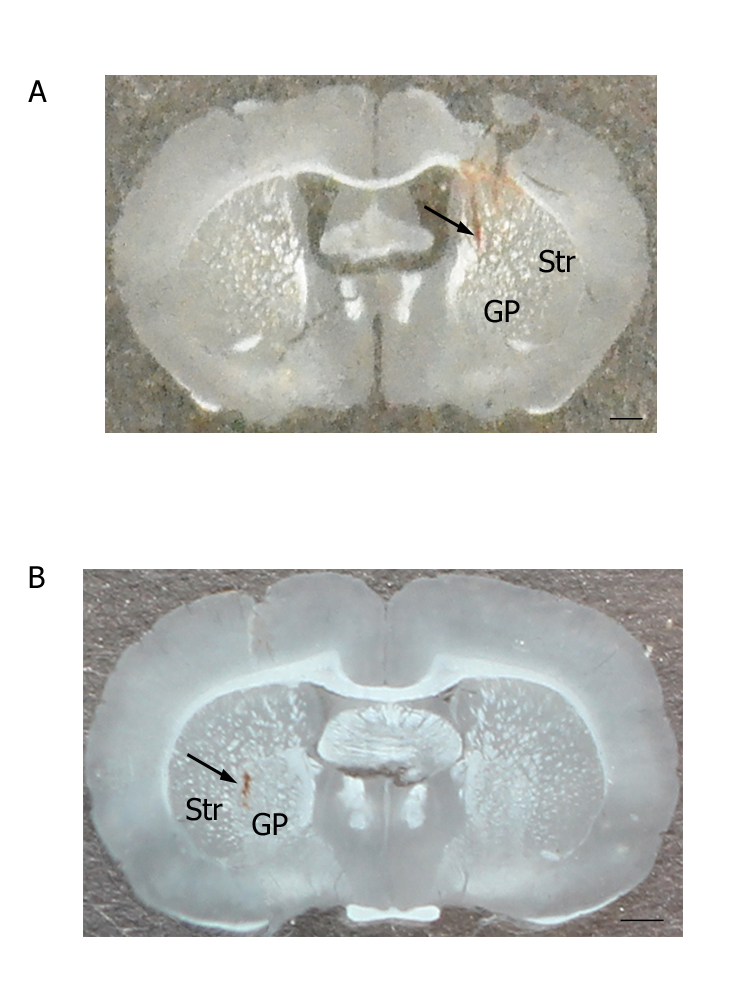

Supplement: FIGURE S2 — Histological verification of the recording sites. Typical photomicrographs of the coronal sections revealing the traces of microelectrodes (arrow pointing) were confined to the globus pallidus in mice (A) and rats (B). Str, striatum; GP, globus pallidus. Scale bars: 500 μm (A), 1 mm (B). [file Image_2.TIF]
